# Association of Systemic Immune Inflammation Index with Estimated Pulse Wave Velocity, Atherogenic Index of Plasma, Triglyceride-Glucose Index, and Cardiovascular Disease: A Large Cross-Sectional Study

**DOI:** 10.1155/2023/1966680

**Published:** 2023-02-15

**Authors:** Shengjue Xiao, Xiaotong Wang, Gang Zhang, Mingyue Tong, Jian Chen, Yufei Zhou, Qian Ji, Naifeng Liu

**Affiliations:** ^1^Department of Cardiology, Zhongda Hospital, School of Medicine, Southeast University, 87 Dingjiaqiao, Nanjing, Jiangsu 210009, China; ^2^Department of Cardiology, The Affiliated Hospital of Xuzhou Medical University, Xuzhou, Jiangsu 221000, China; ^3^Department of Rehabilitation, The First Affiliated Hospital of Anhui Medical University, Anhui Public Health Clinical Center, Hefei, Anhui 230000, China; ^4^Department of Cardiology, Shanghai Institute of Cardiovascular Diseases, Zhongshan Hospital and Institutes of Biomedical Sciences, Fudan University, 180 Feng Lin Road, Shanghai 200032, China; ^5^Department of Emergency, The Second Hospital of Nanjing, Nanjing University of Chinese Medicine, Nanjing, Jiangsu 210003, China

## Abstract

In the U.S. general population, there is a lack of understanding regarding the association between the systemic immune inflammation (SII) index and estimated pulse wave velocity (ePWV), atherogenic index of plasma (AIP), and triglyceride-glucose (TyG) index and cardiovascular disease (CVD). As a result, the objective of our research was to investigate the association between the SII index and ePWV, AIP, and TyG index and incident CVD. We used the National Health and Nutrition Examination Survey (NHANES) data from 1999 to 2018 to conduct this study. The correlation between the SII index and ePWV, AIP, and TyG index was examined using generalized additive models with smooth functions. In addition, the association between SII index and triglyceride (TC), high-density lipoprotein cholesterol (HDL-C), and fast glucose (FBG) also were explored. Finally, we further performed multivariable logistic regression analysis, restricted cubic spline (RCS) plots, and subgroup analysis to study the connection between the SII index and CVD. Our analysis included 17389 subjects from the NHANES database. A substantial positive association existed between SII, WV, and the TyG index. In addition, with the increase of the SII index, AIP showed a trend of decreasing first, then rising, and then decreasing. The SII index was inversely and linearly associated with triglyceride (TG), while positively and linearly associated with fast glucose (FBG). However, high-density lipoprotein cholesterol (HDL-C) had a tendency of first declining, then climbing, and finally falling with the rise in the SII index. After adjusting for potential confounders, compared with the lowest quartiles, the odds ratios with 95% confidence intervals for CVD across the quartiles were 0.914 (0.777, 1.074), 0.935 (0.779, 1.096), and 1.112 (0.956, 1.293) for SII index. The RCS plot showed an inverse U-shaped curve relationship between the SII index and CVD. Overall, this study found a strong correlation between a higher SII index and ePWV and the TyG index. Additionally, these cross-sectional data also revealed a U-shaped connection between the SII index and CVD.

## 1. Introduction

It is characteristic of metabolic syndrome and cardiovascular disease (CVD) that there is systemic inflammation [1]. Systemic inflammation caused by metabolic or immunological conditions is strongly linked to cardiovascular disease [2]. The systemic immune inflammation (SII) index is a measure of systemic inflammation based on the neutrophil-to-lymphocyte ratio and platelet-to-lymphocyte ratio [3]. There is a correlation between changes in gene expression in peripheral blood cells and various forms of systemic inflammation and immunosuppression, such as cardiovascular disease [4]. A recent study shows major cardiovascular events after coronary intervention were better predicted by the SII index than traditional risk factors in coronary artery disease (CAD) patients [5].

Pulse wave velocity (PWV) is a good indicator of arterial stiffness [6]. However, recently, a study found that an equation generated from the reference values for arterial stiffness collaboration can be used to calculate estimated pulse wave velocity (ePWV) using age and mean blood pressure, and the predicted values are similar to those of carotid–femoral pulse wave velocity (cfPWV) [7]. Vlachopoulos et al. published in JAMA calculated an index of cfPWV based on age and mean blood pressure and found that ePWV predicted CVD outcomes independent of traditional CVD risk factors in patients at increased risk for CVD in SPRINT (Systolic Blood Pressure Intervention Trial) [8]. CVD is independently connected with the atherogenic index of plasma (AIP), which is the logarithm of the ratio of triglyceride to high-density lipoprotein cholesterol (TG/HDL-C) concentrations in molar units [9]. Fernández-Macías et al. found that the AIP may be suggested as a valuable and potential biomarker in the early diagnosis of CVD events in developing countries of CVD events in developing countries [10]. The fasting blood glucose (FBG) and TG levels are used to calculate the TyG index, which has been suggested as a reliable indicator of insulin resistance [11]. The risk of arterial stiffness was increased in those with a higher TyG index [12]. Ding et al. also revealed that the incidence of atherosclerotic cardiovascular diseases, CAD, and stroke in persons may all be independently correlated with a higher TyG index [13]. In addition, the TyG index has been linked positively to the risk of CVD in several research, including those on arterial stiffness, carotid atherosclerosis, hypertension, metabolic syndrome, systematic CAD, and coronary artery calcification [14–20].

Recently, the relationship between the SII index and ePWV, AIP, TyG index, and the incidence of CVD is unclear. We hypothesized that the SII index may induce changes in the ePWV, AIP, and TyG indices, which would therefore result in the development of CVD. The objective of this study was to determine the relationship between the SII index and ePWV, AIP, and TyG index. In addition, we investigated further the association between the SII index and incident CVD.

## 2. Material and Methods

### 2.1. Study Population

The current cross-sectional research was based on the NHANES, a survey of nutrition and health in the United States that is representative nationwide [21]. In the total sample of 106203 participants, there were 11312 without SII index data. Furthermore, we excluded participants who did not have ePWV, AIP, and TyG index data (*n* = 65577). In addition, those lacking data on CVD (*n* = 6839) were also excluded. Finally, we also removed the patient's missing demographic and biochemical data to ensure the accuracy of the results (*n* = 5086). A total of 17389 participants were included in the final analysis. The NHANES website (https://www.cdc.gov/nchs/nhanes/) has comprehensive information on the survey's design, methodology, and statistics. The National Center for Health Statistics Research Ethics Review Board approved all protocols, and informed consent was obtained from all participants included in the investigation [22].

### 2.2. Calculation of the SII Index

Blood samples were collected from fasting participants in the study. The automated hematology analyzing devices (Coulter® DxH 800 analyzer) was used to measure blood count (neutrophil, lymphocyte, and platelet counts). In this study, we calculated SII index for each participant as follows: SII index (×10^9^/L) = neutrophil count (×10^9^/L)/lymphocyte count (×10^9^/L) × platelet count (×10^9^/L) [23]. Furthermore, SII index was categorized into quartiles: Q1 (1.529-330.179), Q2 (330.180-463.273), Q3 (463.274-654.615), and Q4 (654.616-28397.276).

### 2.3. The ePWV, AIP, TyG Index, and CVD Measurement

The ePWV was calculated using an equation incorporating age and mean arterial pressure and expressed as m/s [24]. The AIP was determined by analyzing a blood sample and using the following formula: Log10 (TG (mg/dL)/HDL − C (mg/dL)) [25]. In addition, the TyG index was calculated as ln(fasting TG (mg/dL) × FBG (mg/dL)/2) [26]. The outcome of CVDs was defined as 5 self-reported CVDs end point, which included coronary heart disease (CHD), congestive heart failure (CHF), angina pectoris, heart attack, and stroke [27]. The subject was recorded as having CVDs if she/he answered “yes” to the following question: “Has a doctor or other health professional ever told you that you had congestive heart failure/coronary heart disease/angina pectoris/heart attack/stroke?” with standardized medical status questionnaires used in individual interviews. More questionnaire data details are available in (https://www.cdc.gov/nchs/data/nhanes/nhanes_13_14/1999-2018_overview_brochure.pdf).

### 2.4. Covariates

The following covariates were considered in the study: age, gender, race/ethnicity, family poverty income ratio (PIR), education level, marital status, history of hypertension, and diabetes mellitus (DM), smoker, drinker, body mass index (BMI), waist circumference, the history of hyperlipidemia, Charlson comorbidity index (CCI), hemoglobin (Hb), fast glucose (FBG), high-density lipoprotein cholesterol (HDL-C), total cholesterol (TC), triglyceride (TG), and insulin resistance index (HOMA-IR). Hb, HDL-C, TC, TG, FBG, and HOMA-IR were all determined in the laboratory. More information regarding the variables used in this study is available at https://www.cdc.gov/nchs/nhanes/index.htm.

### 2.5. Statistical Analysis

Mean (standard deviation) and quantity (percentage, %) are used to represent continuous and categorical variables, respectively. For continuous variables, the weighted linear regression model was used. In addition, to compare the constituent ratios between each group, the weighted chi-square test was performed. Generalized additive models with smooth functions were used to examine the correlation between the SII index and ePWV, AIP, and TyG index. In addition, the association between SII index and triglyceride (TC), high-density lipoprotein cholesterol (HDL-C), and fast glucose (FBG) also were explored. Multivariate logistic regression analysis with a restricted cubic spline (RCS) plot was performed to explore the potential nonlinearity of the association between DII and CVD. Subgroup analysis stratified by age, gender, race/ethnicity, hypertension, DM, and obesity was applied to examine the association between the SII index and CVD. First, model 1 was adjusted for age and gender. Second, model 2 was further adjusted for race/ethnicity, education level, marital status, family PIR, hypertension, DM, smoker, and drinker. Finally, model 3 was further adjusted for BMI, waist circumference, the history of hyperlipidemia, Hb, FBG, HDL-C, TC, TG, and HOMA-IR as our core model. The receiver operating characteristic (ROC) curve analysis was used to evaluate the discrimination for CVD, and the Delong test was conducted to compare whether the discrimination was statistically significant. All statistical analyses were performed using R version 3.6.4 (R Foundation for Statistical Computing, Vienna, Austria), Stata version 13.0 (Stata Corporation, College Station, TX, USA), and SPSS version 22.0 (SPSS Inc., Chicago, IL, USA). *P* value < 0.05 was regarded as statistically significant.

## 3. Results

### 3.1. Characteristics of Participants by SII Index Quartiles

The SII index was divided into quartiles, with Q1 serving as the reference group, to further explore the relationship between DII and ePWV, AIP, and the risk of CVD (Table 1). Age, gender, race/ethnicity, family PIR, education level, marital status, smoker, alcohol user, history of hypertension, DM, CHD, CHF, heart attack, stroke, hyperlipidemia, BMI, waist circumference, Hb, FBG, HDL-C, TC, TG, ePWV, AIP, HOMA IR, and TyG index had significant difference among the Q1, Q2, Q3, and Q4 groups. The Q4 group occupied the highest proportion of hypertension, DM, CHD, CHF, angina pectoris, heart attack, stroke, and CVD. In addition, individuals in the Q4 group seem to be the oldest, with 13.8% of them females. Participants in the Q1 group had the lowest BMI, waist circumference, FBG, TC, TG, ePWV, AIP, HOMA IR, and TyG index and the highest level of Hb. Compared with the Q1, Q3, and Q4 groups, individuals in the Q2 group had the highest proportion of heart attack and the highest family PIR. Additionally, participants in the Q3 group had the highest proportion of angina pectoris, and with higher education, compared with the Q1, Q2, and Q4 groups.

### 3.2. Association between SII Index and ePWV, AIP, and TyG Index

**Figure 1:**
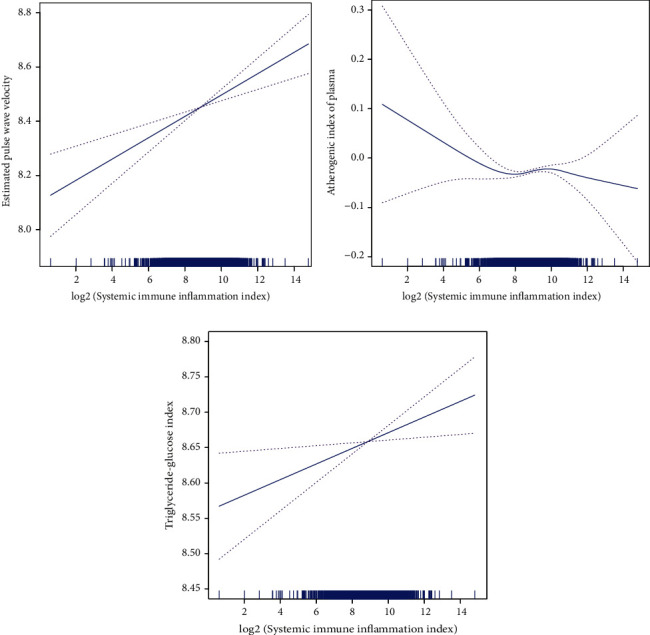
The generalized additive models of the association between SII index with (a) estimated pulse wave velocity, (b) atherogenic index of plasma, and (c) triglyceride-glucose index. Abbreviation: SII: systemic immune inflammation.

**Figure 2:**
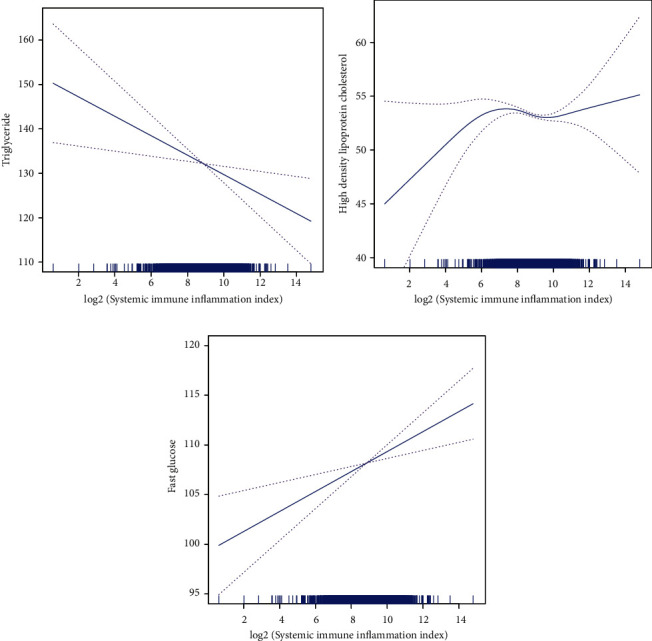
The generalized additive models of the association between SII index with (a) triglyceride, (b) high-density lipoprotein cholesterol, and (c) fast glucose. Abbreviation: SII: systemic immune inflammation.

According to Figures 1(a) and 1(c) , the generalized additive models with smooth functions illustrates the linear association between SII index and ePWV and TyG index.  Figure 1(b) depicts the AIP decreased first, then increased, and finally decreased as SII index increased. The trend of HDL-C increased initially, then decreased, and finally increased with the increase of the SII index (Figure 2(b)). In addition, a negative and linear relationship was found between the SII index and TG, while a positive and linear relationship was found between the SII index and FBG (Figures 2(a), and 2(c)).

### 3.3. Association between SII Index and Total CVD and Individual CVDs

**Figure 3:**
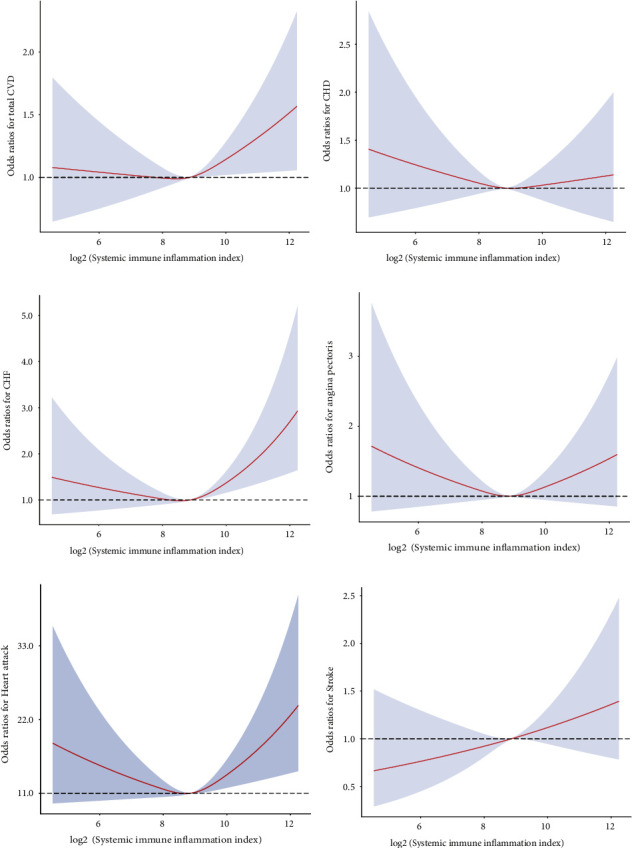
The restricted cubic spline plot of the association between SII index with (a) total CVD, (b) CHD, (c) CHF, (d) angina pectoris, (e) heart attack, and (f) stroke. Abbreviation: SII: systemic immune inflammation; CVD: cardiovascular disease; CHD: coronary heart disease; CHF: congestive heart failure.

Results of the multivariate logistic regression analysis of the SII index and total CVD and individual CVDs are presented in Tables 2 and 3, respectively. After adjusting for underlying confounding variables, the odd ratios (ORs) with 95 percent confidence intervals (CIs) for total CVD across the quartiles were 0.914 (0.777, 1.074), 0.935 (0.779, 1.096), and 1.112 (0.956, 1.293) for the SII index, compared to the Q1 group. In addition, compared with the Q1 group, the fully adjusted ORs and CIs of individual CVDs, including CHD, CHF, angina pectoris, heart attack, and stroke, in the highest quartiles were 1.026 (0.818, 1.287), 1.243 (0.962, 1.605), 1.042 (0.805, 1.349), 1.120 (0.902, 1.391), and 1.263 (0.999, 1.595), respectively. There was a significant association between CHF, angina pectoris, as well as heart attack, and SII index (*P* for trend < 0.05). The restricted cubic spline (RCS) plot is shown in Figure 3(a), representing a U-shaped curve correlation between the SII index and total CVD. In addition, there was a U-shaped curve relationship between the SII index and CHD and heart attack (Figures 3(b)–3(e)); *P* for nonlinearity < 0.05). The RCS plot also revealed the positive and linear association between the SII index and stroke (Figure 3(f); *P* for nonlinearity > 0.05).

### 3.4. ROC Curve Analysis

**Figure 4:**
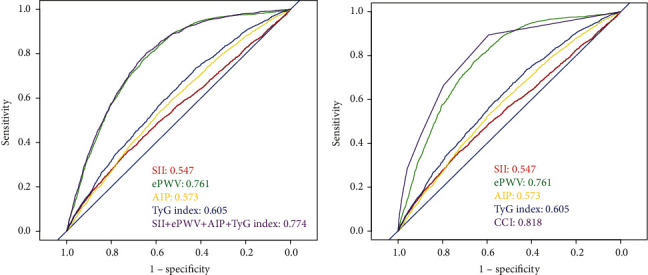
ROC curve analysis was used to test the predictive ability of (a) SII index, ePWV, AIP, and TyG index and their combinations and (b) SII index, ePWV, AIP, TyG index, and CCI index to CVD. Abbreviation: SII: systemic immune inflammation; ePWV: estimated pulse wave velocity; AIP: atherogenic index of plasma; TyG: triglyceride-glucose; CVD: cardiovascular disease.

The predictive value of the SII index, ePWV, AIP, and TyG index for cardiovascular disease risk was analyzed using the ROC curve. ROC analysis showed that the area under the curve (AUC) was 0.547 (95% CI, 0.534-0.561), 0.761 (95% CI, 0.751-0.774), 0.573 (95% CI, 0.560-0.587), 0.605 (95% CI, 0.593-0.619), and 0.774 (95% CI, 0.761-0.783) for the SII index, ePWV, AIP, and TyG index, and the above four indicators, respectively (Figure 4(a)). In addition, ROC analysis was used to evaluate the TyG index and CCI, and the Delong test was used to compare the discrimination between the TyG index and CCI. The AUC of TyG was statistically different from that of CCI (0.605 vs. 0.818, Figure 4(b)).

### 3.5. Subgroup Analysis

Age, gender, race/ethnicity, hypertension, DM, and obesity were stratified analyses that confirmed positive associations between the SII index and total CVD (Table 4). There was a relationship between the SII index and CVD among individuals who were aged ≥60 years and were Mexican American. In addition, the interaction of the SII and CVD with age, gender, race/ethnicity, hypertension, and DM was significant (*P* for interaction < 0.05).

## 4. Discussion

Our study found that the SII index was linearly positively correlated with ePWV, FBG, and TyG index and linearly negatively correlated with TG. The trend of AIP decreased at first, then leveled off, and then decreased with the increase of SII index. As for HDL-C, with the increase of SII index, it first rose to a plateau and then rose again. In addition, with the increase of SII index, the risk of stroke also basically increased linearly, and the risk of individual CVDs decreased first and then increased with the increase of SII index.

The pulse wave propagates with increasing velocity, usually along the aorta to the larger artery and then to the smaller artery. Acute inflammation is thought to reduce wave reflection through peripheral vascular dilation [28]; there are also studies demonstrating that acute inflammation does not alter the ePWV [29]. The ePWV and SII index are linearly and positively correlated, which is inconsistent with previous studies because the SII index not only reflects acute inflammation, which suggests that different inflammation can cause different changes in ePWV, and further experimental exploration is needed for inflammation and ePWV. Coronary arteriosclerosis is closely associated with inflammation; meanwhile, TG and DM are both cardiovascular risk factors, and the SII index is often combined with traditional risk factors to predict cardiovascular events, but few studies have directly demonstrated the relationship between the SII index and TG and FBG [30–32]. In our study, both FBG and TyG index were linearly positively correlated with the SII index, and TG was linearly negatively correlated with the SII index, which is an interesting finding because, from the way of calculation, it seems that TG should have the same trend as FBG and TyG index. We considered that it was caused by the fluctuation of blood glucose level, because compared with TG, FBG is more likely to change in a short period due to the individual's environment and other influences, which may be the potential reason for the different trend of TG. The previous experiment has shown that inflammatory indicators in rheumatoid patients are closely related to dyslipidemia, and some inflammatory factors can be used as predictors of AIP, which is consistent with our findings on the SII index and AIP [33]. However, nearly half of the rheumatoid patients in the experiment had a decrease in HDL-C level, but our study showed that HDL-C decreased with the increase of the SII index. This may be due to differences in the population and suggests that the changing trend of blood lipids is different in different inflammatory diseases.

In recent years, many studies have confirmed the predictive efficacy of the SII index in cardiovascular disease [34–38], and a recent meta-analysis also showed that people with high SII index levels have a significantly increased risk of CVD [39]. The SII index value also increased significantly at different CVD initiations. Our study showed that up to a certain level of SII, individual CVDs increased with increasing SII index, but up to this threshold, CVDs risk decreased with increasing SII index. It is worth noting that within the range near this threshold, the trend of AIP and HDL-C is also stable, and the SII index value in this range is accompanied by a low risk of CVDs. However, considering the calculation method of the SII index, the SII index value may still be in this range in the case of some abnormal blood cell counts, which should be paid special attention to in clinical practice. In addition, the relationship between stroke and the SII index is different from other individual CVDs, which is a positive linear correlation, suggesting that the SII index may be more advantageous in predicting stroke.

Considering the differences in population characteristics, we further adjusted variables for subgroup analysis, and the results showed that the relationship between the SII index and the risk of CVDs was more significant in people who were older, female, Mexican American, and complicated with hypertension. For these people, the SII index may be more accurate in predicting the occurrence of CVDs. DM and BMI did not affect the relationship between the SII index and CVDs, which may be caused by the corresponding changes in blood cells caused by nutritional level. The relationship between the SII index and these two needs to be explored by more studies.

ROC curves were drawn to further verify the predictive efficacy of each indicator on CVDs, and the results showed that ePWV had the most significant effect. ePWV can well reflect the degree of arteriosclerosis, which may be the reason why it has good predictive value in the outcome of various CVDs [7]. It is important to note that although AIP also reflects arteriosclerosis, its predictive efficacy is not as good as ePWV because its calculation depends on lipid levels, and the use of some lipid-lowering agents may interfere with the results. Studies have shown that immune and inflammatory responses play an important role in the development of atherosclerosis, so the SII index has been studied to predict CVDs [40]. However, our results show that the SII index has poor predictive efficacy, which may be due to the combination of other inflammatory and pathological processes, which interferes with the predictive effect of the SII index. Markers calculated by hematological indicators, including AIP, SII index, and TyG index, are susceptible to the interference of accompanying diseases and drug use, which may be the reason why their predictive efficacy is lower than ePWV. In addition, concomitant diseases showed the most significant efficacy in predicting CVDs, suggesting that concomitant diseases are still important risk factors for CVDs and have a more reliable predictive role in the development of CVDs than the above markers. Nevertheless, Spetko et al. found in a cross-sectional study that higher levels of ePWV as an indicator of arterial stiffness were associated with poorer left ventricular diastolic function [41]. In a case-control study, AIP and TyG index helped to predict CVD risk compared to traditional measures [42]. Karadeniz et al. also found that a higher TyG index was strongly associated with major cardiovascular adverse events [43]. Another large prospective study found a significant association between a large increase in the SII index and the risk of CVDs [44]. Our study also showed that the combination of the above markers still had good efficacy in predicting CVDs, and reference to these markers can better assess the risk of CVDs in patients. Studies on predictors of CVDs are ongoing, and a recent Korean study showed that apolipoprotein B has a greater impact on CVDs risk than low-density lipoprotein cholesterol (LDL-C) and non-HDL-C [45]. In another study, small dense low-density lipoprotein cholesterol and low-density lipoprotein particles were also associated with CHD [46]. All the above indexes have the potential to be markers for predicting CVDs. In addition, neutrophil-lymphocyte ratio and platelet-lymphocyte ratio have also been confirmed in many studies to be related to the prognosis of heart disease, so whether they can be used to predict CVDs needs further research [47].

Our study also has some limitations that must be mentioned. First, this is an observational study, which carries a risk of bias. Second, although we adjusted for covariates, there was still potential bias due to individual differences. Third, our study population is in the United States, and further studies are needed to confirm whether it is applicable to other regions.

## 5. Conclusion

In U.S. general population, the SII index and ePWV and TyG index were shown to have a monotonically rising association. In addition, as SII index increased, AIP decreased, then increased, and then decreased again. The SII index displayed a U-curve relationship with CVD, including CHD, CHF, angina pectoris, and heart attack, while the SII index was positively associated with stroke.

## Figures and Tables

**Table 1:** Distribution of baseline characteristics of NHANES cohort across quartiles of SII index.

SII index	Overall	Q1	Q2	Q3	Q4	*P* value
Age (years)	47.100 ± 0.245	45.735 ± 0.384	46.091 ± 0.383	47.674 ± 0.356	48.718 ± 0.352	<0.001
Gender (%)						<0.001
Male	8883 (51.1%)	2469 (14.2%)	2327 (13.4%)	2136 (12.3%)	1951 (11.2%)	
Female	8506 (48.9%)	1879 (10.8%)	2020 (11.6%)	2210 (12.7%)	2397 (13.8%)	
Race (%)						<0.001
Mexican American	3008 (17.3%)	687 (4.0%)	828 (4.8%)	799 (4.6%)	694 (4.0%)	
Other Hispanic	1374 (7.9%)	314 (1.8%)	370 (2.1%)	360 (2.1%)	330 (1.9%)	
Non-Hispanic black	3325 (19.1%)	1301 (7.5%)	771 (4.4%)	672 (3.9%)	581 (3.3%)	
Non-Hispanic white	8264 (47.5%)	1584 (9.1%)	2006 (11.5%)	2204 (12.7%)	2470 (14.2%)	
Other race	1418 (8.2%)	462 (2.7%)	372 (2.1%)	311 (1.8%)	273 (1.6%)	
Family PIR	3.065 ± 0.031	3.016 ± 0.042	3.177 ± 0.044	3.103 ± 0.039	2.959 ± 0.044	<0.001
Education level (%)						<0.001
Less than high school	4394 (25.3%)	1060 (6.1%)	1105 (6.4%)	1126 (6.5%)	1103 (6.3%)	
High school	1513 (8.7%)	484 (2.8%)	381 (2.2%)	304 (1.7%)	344(7.9%)	
More than high school	11482 (66.0%)	2804 (16.1%)	2861 (16.5%)	2916 (16.8%)	2901 (16.7%)	
Marital status (%)						<0.001
Having a partner	10727 (61.7%)	2689 (15.5%)	2790 (16.0%)	2725 (15.7%)	2523 (14.5%)	
No partner	3723 (21.4%)	817 (4.7%)	849 (4.9%)	904 (5.2%)	1153 (6.6%)	
Unmarried	2939 (16.9%)	842 (4.8%)	708 (4.1%)	717 (4.1%)	672 (3.9%)	
Smoker (%)						<0.001
No	9210 (53.0%)	2472 (14.2%)	2408 (13.8%)	2294 (13.2%)	2036 (11.7%)	
Former	4495 (25.8%)	1060 (6.1%)	1131 (6.5%)	1109 (6.4%)	1195 (6.9%)	
Now	3648 (21.2%)	816 (4.7%)	808 (4.6%)	943 (5.4%)	1117 (6.4%)	
Alcohol user (%)						<0.001
Never	2296 (13.2%)	601 (3.5%)	578 (3.3%)	586 (3.4%)	531 (3.1%)	
Former	3008 (17.3%)	708 (4.1%)	697 (4.0%)	728 (4.2%)	875 (5.0%)	
Mild	6001 (34.5%)	1566 (9.0%)	1498 (8.6%)	1501 (8.6%)	1436 (8.3%)	
Moderate	2583 (14.9%)	646 (3.7%)	663 (3.8%)	635 (3.7%)	639 (3.7%)	
Heavy	3501 (20.1%)	827 (4.8%)	911 (5.2%)	896 (5.2%)	867 (5.0%)	
Hypertension (%)						<0.001
No	9899 (56.9%)	2583 (14.9%)	2616 (15.0%)	2456 (14.1%)	2244 (12.9%)	
Yes	7490 (43.1%)	1765 (10.2%)	1731 (10.0%)	1890 (10.9%)	2104 (12.1%)	
DM (%)						<0.001
No	14160 (81.4%)	3616 (20.8%)	3551 (20.4%)	3569 (20.5%)	3424 (19.7%)	
Yes	3229 (18.6%)	732 (4.2%)	796 (4.6%)	777 (4.5%)	924 (5.3%)	
CHD (%)						0.037
No	16682 (95.9%)	4190 (24.1%)	4196 (24.1%)	4170 (24.0%)	4126 (23.7%)	
Yes	707 (4.1%)	158 (0.9%)	151 (0.9%)	176 (1.0%)	222 (1.3%)	
CHF (%)						<0.001
No	16886 (97.1%)	4240 (24.4%)	4252 (24.5%)	4231 (24.3%)	4163 (23.9%)	
Yes	503 (2.9%)	108 (0.6%)	95 (0.5%)	115 (0.7%)	185 (1.1%)	
Angina (%)						0.180
No	16907 (97.2%)	4237 (24.4%)	4230 (24.3%)	4249 (24.4%)	4191 (24.1%)	
Yes	482 (2.8%)	111 (0.6%)	117 (0.7%)	97 (0.6%)	157 (0.9%)	
Heart attack (%)						<0.001
No	16671 (95.9%)	4182 (24.0%)	4203 (24.2%)	4188(24.1%)	4098 (23.6%)	
Yes	718 (4.1%)	166 (1.0%)	144 (0.8%)	158 (0.9%)	250 (1.4%)	
Stroke (%)						0.001
No	16765 (96.4%)	4221 (24.3%)	4210 (24.2%)	4197 (24.1%)	4137 (23.8%)	
Yes	624 (3.6%)	127 (0.7%)	137 (0.8%)	149 (0.9%)	211 (1.2%)	
Hyperlipidemia (%)						<0.001
No	4666 (26.8%)	1357 (7.8%)	1180 (6.8%)	1051 (6.0%)	1078 (6.2%)	
Yes	12723 (73.2%)	2991 (17.2%)	3167 (18.2%)	3295 (18.9%)	3270 (18.8)	
CVD (%)						<0.001
No	15562 (89.5%)	3936 (22.6%)	3692 (22.8%)	3906 (22.5%)	3458(21.6%)	
Yes	1827 (10.5%)	412 (2.4%)	385 (2.2%)	440 (2.5%)	590 (3.4%)	
BMI (kg/m^2^)	28.669 ± 0.080	27.755 ± 0.124	28.365 ± 0.137	28.962 ± 0.128	29.478 ± 0.149	<0.001
Waist circumference (cm)	98.440 ± 0.203	95.907 ± 0.324	97.704 ± 0.349	99.427 ± 0.331	100.397 ± 0.357	<0.001
Hb (g/dL)	14.473 ± 0.023	14.478 ± 0.031	14.565 ± 0.034	14.523 ± 0.031	14.326 ± 0.033	<0.001
FBG (mg/dL)	104.961 ± 0.329	103.250 ± 0.501	104.710 ± 0.600	105.246 ± 0.538	106.419 ± 0.573	<0.001
HDL-C (mg/dL)	53.594 ± 0.207	54.798 ± 0.375	53.808 ± 0.383	52.996 ± 0.357	52.929 ± 0.321	<0.001
TC (mg/dL)	195.503 ± 0.502	192.964 ± 0.935	196.802 ± 0.793	197.124 ± 0.784	194.804 ± 0.785	0.001
TG (mg/dL)	131.633 ± 1.148	125.302 ± 2.315	130.043 ± 2.036	135.225 ± 1.989	135.139 ± 2.236	0.003
CCI	0.87 ± 0.02	0.81 ± 0.03	0.82 ± 0.03	0.84 ± 0.02	1.02 ± 0.03	<0.001
ePWV	8.052 ± 0.026	7.898 ± 0.038	7.939 ± 0.042	8.095 ± 0.039	8.254 ± 0.040	<0.001
AIP	−0.033 ± 0.004	−0.077 ± 0.008	−0.039 ± 0.007	−0.009 ± 0.007	−0.011 ± 0.006	<0.001
HOMA IR	3.511 ± 0.053	3.242 ± 0.083	3.363 ± 0.089	3.592 ± 0.087	3.811 ± 0.101	<0.001
TyG index	8.629 ± 0.008	8.532 ± 0.015	8.615 ± 0.013	8.674 ± 0.013	8.680 ± 0.013	<0.001

Abbreviations: Q1: 1.529-330.179; Q2: 330.180-463.273; Q3: 463.274-654.615; Q4: 654.616-28397.276; NHANES: National Health and Nutrition Examination Survey; SII: systemic immune inflammation; PIR: poverty income ratio; DM: diabetes mellitus; CHD: coronary heart disease; CHF: congestive heart failure; CVD: cardiovascular disease; BMI: body mass index; Hb: hemoglobin; FBG: fast glucose; HDL-C: high-density lipoprotein cholesterol; TC: total cholesterol; TG: triglyceride; CCI: Charlson comorbidity index; ePWV: estimated pulse wave velocity; AIP: atherogenic index of plasma; HOMA IR: insulin resistance index; TyG: triglyceride-glucose.

**Table 2:** Adjusted ORs for associations between SII index and the incidence of total CVD.

SII index	Model 1	Model 2	Model 3
	OR (95% CI)	OR (95% CI)	OR (95% CI)
Q1	Ref.	Ref.	Ref.
Q2	0.920 (0.789, 1.074)	0.911 (0.777, 1.067)	0.914 (0.777, 1.074)
Q3	0.997 (0.858, 1.159)	0.965 (0.826, 1.126)	0.935 (0.779, 1.096)
Q4	1.270 (1.100, 1.466)	1.153 (0.995, 1.336)	1.112 (0.956, 1.293)
*P* for trend	<0.001	0.012	0.043

Abbreviations: Q1: 1.529-330.179; Q2: 330.180-463.273; Q3: 463.274-654.615; Q4: 654.616-28397.276; SII: systemic immune inflammation; OR: odd ratio; CI: confidence interval. Model 1 was adjusted for age and gender. Model 2 was further adjusted for education level, marital status, family poverty income ratio, hypertension, diabetes mellitus, smoker, and drinker. Model 3 was further adjusted for body mass index, waist circumference, hyperlipidemia, hemoglobin, fast glucose, high-density lipoprotein cholesterol, total cholesterol, triglyceride, and insulin resistance index.

**Table 3:** Adjusted ORs for associations between SII index and individual CVDs.

SII index	CHD	CHF	Angina	Heart attack	Stroke
	OR (95% CI)	OR (95% CI)	OR (95% CI)	OR (95% CI)	OR (95% CI)
Q1	1.00	1.00	1.00	1.00	1.00
Q2	0.934 (0.732, 1.192)	0.875 (0.654, 1.170)	1.032 (0.786, 1.356)	0.865 (0.680, 1.100)	1.097 (0.852, 1.413)
Q3	0.969 (0.765, 1.226)	0.928 (0.702, 1.227)	0.731 (0.549, 0.973)^∗^	0.823 (0.650, 1.043)	1.039 (0.810, 1.333)
Q4	1.026 (0.818, 1.287)	1.243 (0.962, 1.605)	1.042 (0.805, 1.349)	1.120 (0.902, 1.391)	1.263 (0.999, 1.595)
*P* for trend	0.869	0.031	0.040	0.022	0.181

Abbreviations: Q1: 1.529-330.179; Q2: 330.180-463.273; Q3: 463.274-654.615; Q4: 654.616-28397.276; SII: systemic immune inflammation; CVD: cardiovascular disease; CHD: coronary heart disease; CHF: congestive heart failure; OR: odd ratio; CI: confidence interval. Analysis was adjusted for age, gender, education level, race/ethnicity, marital status, family poverty-income ratio, hypertension, diabetes mellitus, smoker, alcohol user, body mass index, body mass index, waist circumference, hemoglobin, glucose, high-density lipoprotein cholesterol, total cholesterol, and triglyceride.

**Table 4:** Subgroup analysis for the associations of SII index with the prevalence of total CVD.

	Q1	Q2	Q3	Q4	*P* for trend	*P* for interaction
	OR (95% CI)	OR (95% CI)	OR (95% CI)	OR (95% CI)		
Age						<0.001
<60	1.00	0.911 (0.746, 1.316)	0.815 (0.610, 1.089)	1.036 (0.789, 1.360)	0.343	
≥60	1.00	0.920 (0.758, 1.118)	1.051 (0.871, 1.268)	1.259 (1.052, 1.507)^∗^	0.005	
Gender						0.005
Male	1.00	0.824 (0.664, 1.023)	0.974(0.790, 1.200)	1.092(0.892, 1.337)	0.068	
Female	1.00	1.050 (0.819, 1.345)	0.892 (0.698, 1.140)	1.135 (0.902, 1.428)	0.194	
Race/ethnicity						0.011
Mexican American	1.00	1.117 (0.699, 1.785)	0.936 (0.580, 1.512)	1.730 (1.096, 2.731)^∗^	0.021	
Other Hispanic	1.00	0.731 (0.397, 1.349)	0.897 (0.448, 1.647)	0.945 (0.525, 1.702)	0.769	
Non-Hispanic Black	1.00	0.838 (0.604, 1.1620	0.787 (0.563, 1.101)	1.073 (0.768, 1.501)	0.295	
Non-Hispanic White	1.00	0.945 (0.746, 1.197)	0.976 (0.779, 1.223)	1.013 (0.817, 1.256)	0.921	
Other race	1.00	1.149 (0.563, 2.345)	1.351 (0.644, 2.835)	2.042 91.024, 4.075)^∗^	0.201	
Hypertension						<0.001
No	1.00	1.009 (0.726, 1.401)	1.147 (0.834, 1.578)	1.229 (0.897, 1.684)	0.507	
Yes	1.00	0.902 (0.749, 1.087)	0.889 (0.741, 1.066)	1.094 (0.921, 1.299)	0.055	
DM						0.033
No	1.00	0.920 (0.749, 1.130)	0.906 (0.742, 1.105)	1.113 (0.921, 1.345)	0.120	
Yes	1.00	0.918 (0.706, 1.195)	0.999 (0.770, 1.298)	1.112 (0.867, 1.426)	0.490	
BMI						0.082
<30 kg/m^2^	1.00	0.935 (0.755, 1.157)	0.943 (0.765, 1.161)	1.154 (0.946, 1.408)	0.123	
≥30 kg/m^2^	1.00	0.907 (0.706, 1.163)	0.943 (0.738, 1.204)	1.064 (0.842, 1.345)	0.544	

Abbreviations: Q1: 1.529-330.179; Q2: 330.180-463.273; Q3: 463.274-654.615; Q4: 654.616-28397.276; SII: systemic immune inflammation; ^∗^*P* < 0.05; OR: odd ratio; CI: confidence interval. Analysis was adjusted for age, gender, race/ethnicity, education level, marital status, family poverty-income ratio, hypertension, diabetes mellitus, smoker, alcohol user, body mass index, waist circumference, hemoglobin, fast glucose, high-density lipoprotein cholesterol, total cholesterol, and triglyceride.

## Data Availability

The survey data are publicly available on the Internet for data users and researchers throughout the world https://www.cdc.gov/nchs/nhanes/.
